# *Shigella flexneri* and *Shigella sonnei* among children under five in Kenya’s urban informal settlement

**DOI:** 10.1186/s13099-026-00824-6

**Published:** 2026-03-22

**Authors:** Christine Kioko, John M. Maingi, Cecilia Mbae, Phelgona Otieno, Collins Kebenei, Winfred Mbithi, Amos Njuguna, Schola Kamwethya, Diana Imoli, Darius Ideke, Jessicah Jepchirchir, Kelvin Kering, Samuel Kariuki

**Affiliations:** 1 Centre for Microbiology Research, Kenya Medical Research Institute, Nairobi, Kenya; 2 Department of Biochemistry, Microbiology and Biotechnology, Kenyatta University, Nairobi, Kenya; 3 Drugs for Neglected Diseases Initiative, Nairobi, Kenya

**Keywords:** *Shigella*, Children, Diarrhoea, Risk factors, Informal settlement

## Abstract

**Background:**

*Shigella* species are a significant global public health threat with the highest burden of *Shigella*-associated diarrhoea in children under five years of age, particularly in low- and middle-income countries. Given the high burden of shigellosis in these settings, there is a need for continuous surveillance to identify the prevalent species, risk factors, and determine appropriate intervention strategies.

**Methods:**

This study aimed to determine *the prevalence of Shigella flexneri (S. flexneri)* and *Shigella sonnei (S. sonnei)*,* assess risk* factors and identify Antimicrobial Resistance (AMR) genes present in samples positive for either species among children under five in Mukuru informal settlement. Between August 2023 and November 2024, 386 children presenting with diarrhoea at any of the four selected health facilities in Mukuru were enrolled. Stool samples or rectal swabs were collected and subjected to TaqMan Polymerase Chain Reaction (TAC PCR) for the detection of *S. flexneri*,* S. sonnei* and selected AMR genes. Case Report Forms (CRF) and structured questionnaires were used to collect medical history, behavioural and socioeconomic data, respectively.

**Results:**

*The prevalence of S. flexneri and S. sonnei* among the 386 participants was 15.54% (60/386). Positivity for *S. flexneri* and *S. sonnei* by age group was highest among children aged 12–23 months (24.84%, 40/161) and lowest among those aged 24–59 months (2.08%, 2/96). *S. flexneri* was the most prevalent species, with serotypes 2 and X being the most detected. Among the samples positive for *S. flexneri* and *S. sonnei*, the *mphA* gene was detected in 98.33% (59/60), the β-lactamase gene *CTX-M1-9* was present in 85% (51/60), and *CTX-M2-8-25* was identified in 26.67% (16/60) of samples. Age (aOR = 1.025, 95% CI: 1.003–1.047, *P* = 0.027) and obtaining vegetables from the market (aOR = 2.737, 95% CI: 1.081–6.930, *P* = 0.034) were significantly associated with shigellosis.

**Conclusions:**

These findings show that shigellosis due to *S. flexneri* and *S. sonnei* remains a significant contributor to the burden of diarrheal disease, particularly among young children. The high prevalence of AMR genes, especially *mphA* and *CTX-M* β-lactamases, indicates widespread resistance to macrolides and extended-spectrum β-lactams. Addressing this challenge may require a multifaceted approach, including enhanced antimicrobial resistance surveillance, strengthened Water, Sanitation, and Hygiene (WASH) interventions, and the potential deployment of *Shigella* vaccines among vulnerable populations.

## Background

Diarrhoea is a leading cause of morbidity and mortality, accounting for 3.6% of the global burden of disease [1]. It is estimated that 1.6 million deaths (all ages) occur worldwide as a result of diarrheal disease [2]. Shigellosis remains a global public health threat, with an estimated 160 million cases reported annually, approximately 60% of which occur in children [3]. Of these cases, around 160,000 die each year. The greatest burden of *Shigella-*associated diarrhoea is among children under five years of age living in low and middle-income countries (LMICs) [4] with an estimated 42,000–94,000 deaths each year [5]. These settings are often characterized by poor sanitation, insufficient hygiene, inadequate safe drinking water, poor health, and nutritional status [6]. A meta-analysis of studies across Africa estimated the pooled prevalence of *Shigella* infection at 5.9%, with Eastern Africa reporting a slightly higher prevalence of 6.2% [7]. In Kenya, the Vaccine Impact on Diarrhoea in Africa (VIDA) study found that *Shigella*-attributable disease accounted for 18.7% of all moderate-to-severe diarrhoea (MSD) cases [8]. Earlier studies in similar Kenyan settings reported *Shigella* incidence of 24% between January 2007 and December 2010, while a more recent investigation documented a markedly lower isolation rate of 1.4% between November 2020 and December 2022 [9, 10].

The primary mode of *Shigella* transmission is the faecal-oral route and the infectious dose is very low (10–100 organisms) [11]. Vulnerable populations include immunocompromised individuals, children under five years, Men having Sex with Men (MSM), the military on deployment and persons from high-income countries travelling to endemic settings [12]. The bacterium is serologically grouped into four species: *Shigella dysenteriae*,* Shigella flexneri*,* Shigella boydii* and *Shigella sonnei* [13] also known as *Shigella* subgroups A, B, C, and D respectively [14]. These subgroups are geographically distributed based on the economic development of countries [15]. The four species are further subdivided into serotypes with *S. flexneri* serotypes 1b, 2a, 3a, 4a, and 6 accounting for most of the shigellosis in developing countries [16].

Rehydration therapy is adequate for management of mild shigellosis, while severe cases require antibiotics to minimize illness duration, severity and transmission [17]. The management of shigellosis has become more challenging due to the emergence of multidrug-resistant (MDR) strains [18], exhibiting resistance to third-generation cephalosporins, azithromycin and fluoroquinolone [19]. Diarrhoea caused by MDR bacteria can result in longer hospitalization, poor treatment outcomes, and increased mortality [20]. The World Health Organization (WHO) has listed *Shigella* among the pathogens for which development of new interventions, such as a safe, effective and affordable vaccine, is a priority [21]. An effective *Shigella* vaccine will not only reduce antibiotic use but also help limit the further emergence of enteric pathogens resistant to antimicrobials [12].

Despite these advances, there is limited recent data on the current prevalence of *Shigella flexneri* and *Shigella sonnei*, circulating serotypes, risk factors, and carriage of AMR genes among children under five years living in urban informal settlements in Kenya. Understanding these epidemiological features is critical for guiding prevention, management, and vaccination strategies. To address this gap, we conducted a study in Mukuru informal settlement using a TaqMan Array Card (TAC)-based real-time polymerase chain reaction (qPCR) assay, which provides high sensitivity, specificity, and rapid turnaround time [22].

## Methods

### Study design and site

This was a cross-sectional study conducted between August 2023 and November 2024. The study was conducted in Mukuru informal settlement, located approximately 20 km southeast of the Nairobi Central Business District (CBD) in Kenya, with an estimated population of 301,683 inhabitants [23]. While Mukuru is divided into 8 villages, this study focused on two villages: Mukuru kwa Njenga and Mukuru kwa Ruben, which have previously been identified as hotspots for diarrheal diseases [10]. The area is characterized by overcrowding, poor sanitary conditions, limited access to safe drinking water, poor waste management, poor hygienic practices, and a predominantly low socioeconomic population [24].

### Sample size calculation

The sample size was calculated using Fisher’s formula, applying a prevalence of 50% as a conservative estimate due to the limited availability of comparable studies in similar settings and the absence of prior PCR-based prevalence data for this population.

n = the sample size,

z = the standard normal deviation which corresponds to 95% confidence interval (= 1.96);

P= prevalence of *Shigella* in children under five years (50%).

d= degree of precision (0.05).

n= $$\frac{{1.96}^{2}\times0.5\left(0.5\right)}{{0.05}^{2}}$$.

*n* = 385.

### Study participant recruitment and sample collection

This study was conducted in accordance with the ethical principles of the Declaration of Helsinki. Children under five years of age presenting with diarrhoea were purposively recruited from four outpatient health facilities serving the Mukuru population: Mukuru Health Centre, Medical Missionaries of Mary, Our Lady of Nazareth, and Reuben Health Centre. Diarrhoea was defined as having ≥ 3 episodes of loose stools in the 24 hours preceding presentation at the health facility. Participants with chronic illnesses, those residing outside the study area, or those whose parents or guardians did not provide consent were excluded from the study. Age was stratified into three categories: 1–11, 12–23, and 24–59 months to reflect key developmental stages and vulnerability patterns in early childhood, as these intervals align with variations in immunity, nutritional needs, and exposure risks. Written informed consent was obtained from the parent or guardian before recruitment, after which a unique identifier was assigned to each participant for identification throughout the study. Fecal samples or rectal swabs were collected in stool containers at the health facility before being aliquoted into cryovials. The samples were then transported in a cooler box to the Centre for Microbiology Research-Kenya Medical Research Institute (CMR-KEMRI) laboratory and stored in -80°C freezers awaiting laboratory processing. Case Report Forms (CRF) were used to collect the medical history of the participants. Demographic, household WASH, and socioeconomic factors were collected through structured questionnaires in the mobile-based Epicollect 5 platform.

## Laboratory analysis

### Total nucleic acid extraction

Total nucleic acid (TNA) was extracted from the fecal samples or rectal swabs using the QIAmp Fast DNA Stool Mini Kit (Qiagen, Hilden, Germany) following the manufacturer’s instructions. For whole stool samples, 200 mg (180–220 mg) or 200 µL if watery, was aliquoted into 2 mL screw cap tubes. The fecal samples were then processed through a lysate preparation procedure involving inhibitor removal using 1mL of InhibitEX buffer, mechanical disruption by bead beating (Glass beads, Sigma G1277-500G), purification and elution of TNA with 200 µL of elution buffer (ATE) using spin columns. To assess extraction and amplification efficiency, external controls— Phocine Herpesvirus (PhHV) and MS2 bacteriophage—were introduced to each sample during the lysate preparation. The extracted TNA was stored at -80°C for subsequent analysis. Additionally, a blank control was included in each extraction batch and processed through the entire protocol to monitor for potential contamination.

### TAC PCR

An enteric TaqMan Array Card (TAC) custom-designed by University of Virginia (UVA), manufactured by ThermoFisher, containing various gene targets listed in Table 1, was used to detect *Shigella* (*S. flexneri* and *S. sonnei*), *S. flexneri* serotypes, and AMR genes. *Shigella dysenteriae* and *Shigella boydii* are rarely detected in this setting, epidemiologically, and were therefore excluded from the TAC panel.

**Table 1:** Quantitative polymerase chain reaction targets used in the enteric TAC panel

Target	Gene target
*Shigella/Enteroinvasive E. coli*	*IpaH*
*S. flexneri 1ab*	*gtrl*,* oac*
*S. flexneri 2*	*gtrll*,* gtrX*
*S. flexneri 3*	*gtrX*,* oac*
*S. flexneri 4*	*gtrlV*,* oac*
*S. flexneri 5*	*gtrV*,* oac*,* gtrX*
*S. flexneri 6*	*wzx6*
*S. flexneri 7a*	*gtrl*,* gtrlc*
*S. flexneri X*	*gtrX*
*S. sonnei*	*Rhs*,* pm*
Macrolide resistance	*ermA*,* ermB*,* ermC*,* mphA*,* mphB*,* mefA*,* msrA*,* msrD*
Polymyxin resistance	*MCR1*
Beta lactam resistance	*CTX-M*,* M1*,*M2*,*M25*,*M9*
MS2	*MS2g1*
PhHv	*gB*

*TAC, Taqman Array Card; MS2, MS2 bacteriophage; PhHv, Phocine Herpesvirus

The master mix was prepared by mixing 425 µL of Ag-Path-ID 2X RT-PCR buffer and 34 µL Ag-Path-ID enzyme mix, then 54 µL was aliquoted into microcentrifuge tubes containing the extracted TNA. For whole fecal samples, 20 µL of TNA extract was supplemented with 26 µL nuclease-free water, while for rectal swabs or extraction blanks, 46 µL of TNA was used. 100 µL of the PCR reaction mix was transferred into the TAC port. The TAC was centrifuged at 1,200 rpm for two consecutive 1-minute spins to resuspend the reactions throughout the card, sealed, and then run on a ViiA7 real-time PCR system (Life Technologies, California, USA). TAC PCR was conducted under programmed conditions (Table 2).

**Table 2:** Programmed TAC PCR cycling conditions for enteric pathogen detection

Step	Temperature	Time
Reverse transcription (1 cycle)	45 °C	20 min
Denaturization (1 cycle)	95 °C	10 min
PCR (40 cycles)	95 °C	15 s
Data collection	60 °C	1 min

*PCR, Polymerase Chain Reaction

Three types of controls were included throughout the testing procedure: TAC positive control, external controls and extraction blank. The positive control contained all targets in a single tube (plasmid for DNA targets and in vitro transcripts for RNA viruses) and also served as standard curve material for quantification analyses. Based on in-house validation, an analytical cutoff cycle threshold (Ct) value of 35 was set for the pathogen targets. Negative results (no amplification or Ct > 35) were considered valid only if the external controls (PHhv and MS2) showed amplification with a Ct < 35. Positive results were validated only when the corresponding extraction blank, processed alongside the sample, exhibited no amplification for the relevant targets.

### Data analysis

Using the QuantStudio real-time PCR software, amplification curves were reviewed for each target individually, and baselines were adjusted as necessary to correct any false-positive or false-negative results, as well as inaccurate Ct values. The results were exported to an Excel file once all targets had been thoroughly reviewed and adjusted as needed. The data was analysed using STATA version 17. Logistic regression analysis was conducted to evaluate the association between each predictor variable and the outcome of interest which is *Shigella* infection. Crude Odds Ratios (CORs) with corresponding 95% confidence intervals were calculated for each variable. Subsequently, multivariable logistic regression was performed, incorporating all variables that were statistically significant in the bivariate analysis. This yielded Adjusted Odds Ratios (AORs) with 95% confidence intervals. The threshold for statistical significance was set at *p* < 0.05 (two-tailed) for all analyses.

## Results

### Detection of *Shigella flexneri* and *Shigella sonnei* among children under five years

A total of 386 children were enrolled in the study, of whom 208 (53.89%) were male and 178 (46.11%) were female. Majority (290, 75.13%) of the participants were aged < 24 months (Table 3). Age distribution showed that among the participants, 161 (41.71%) were between 12 and 23 months and 96 (24.87%) were ≥ 24 months. The overall detection rate of *S. flexneri and S. sonnei* was 15.54% (60/386) (Table 3).

Positivity for *S. flexneri* and *S. sonnei* varied markedly by age: children aged 12–23 months had the highest detection rate at 24.84% (40/161), whereas those aged 24–59 months had the lowest at 2.08% (2/96) (Table 3). Age in months was significantly associated with *S. flexneri and S. sonnei* detection (COR 0.131, 95% CI 0.030–0.58, *p* = 0.007). The combined burden of these species among children below 24 months was (20%, 58/290), tenfold higher than in children ≥ 24 months (2.08%, 2/96). Majority of the participants, 139 (36.01%), were recruited from the Medical Missionaries of Mary Health Facility, while Our Lady of Nazareth had the least participants, with 25 (6.48%) recruited.

**Table 3:** Sociodemographic characteristics of pediatric patients with diarrhoea, urban Kenya

Variable	Categories	Participants (*n*)	Positive F (%)	COR	*p*-value	95% CI
Age in Months	< 12 months	129	18 (13.95)	ref		
	12–23 months	161	40 (24.84)	2.039	0.023	1.104–3.76
	24–59 months	96	2 (2.08)	0.131	0.007	0.030–0.58
Gender	Female	178	32 (17.98)	ref		
	Male	208	28 (13.46)	0.710	0.224	0.409–1.233

*COR, crude odds ratio; CI, confidence interval; ref, reference category

**Fig. 1:**
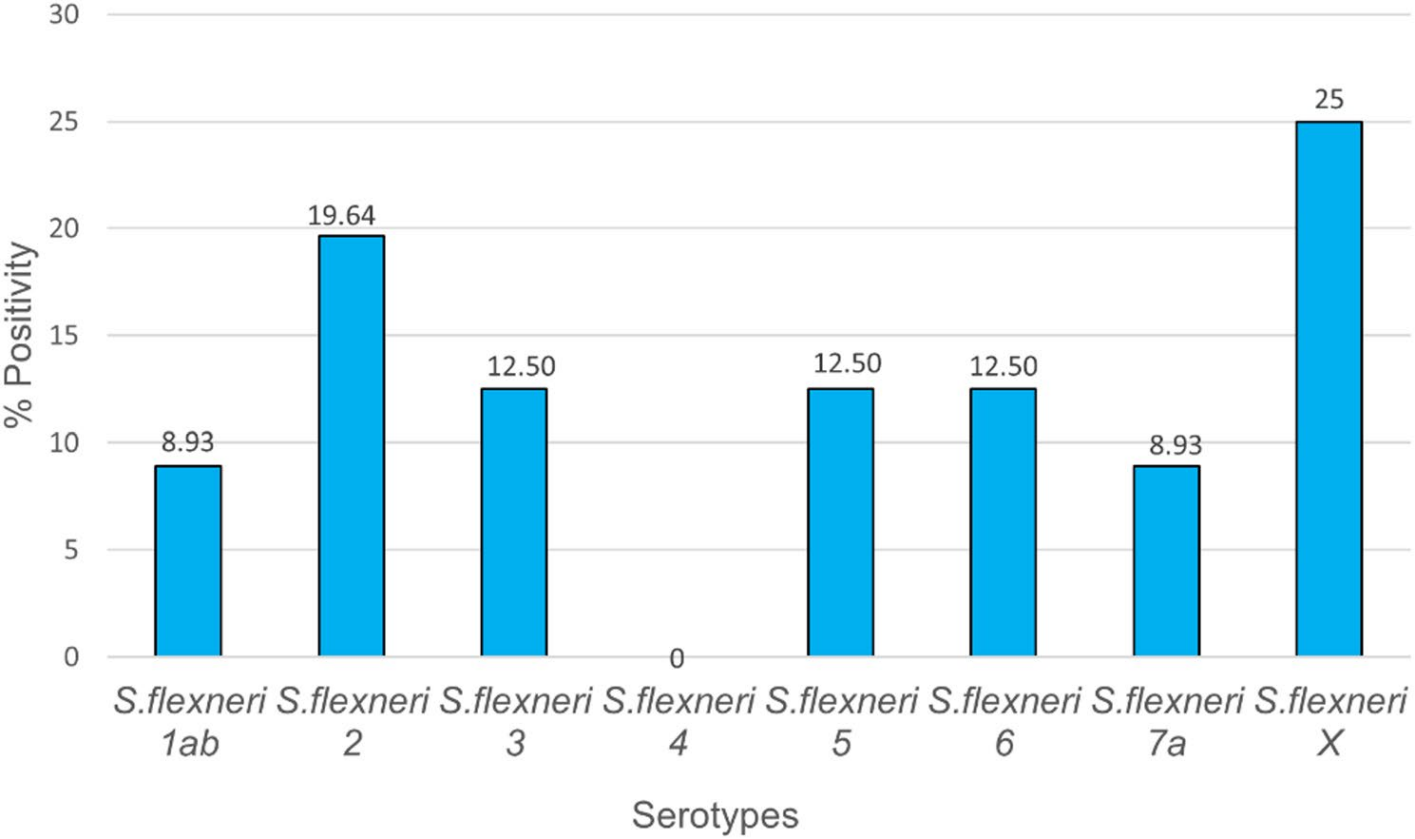
Bar chart showing the proportion of samples positive for each *S. flexneri* serotype. Positivity varied across serotypes, with *S. flexneri* 2 and *S. flexneri* X showing the highest proportions, while *S. flexneri* 4 recorded no detections

Among the 60 samples positive for *S. flexneri* and *S. sonnei*, 55 (91.67%) had a single species (*S. flexneri*) detected, while in 5 (8.33%) samples both *S. flexneri* and *S. sonnei* were detected. Ten of the *S*. *flexneri* positive samples exhibited more than one serotype. *S. flexneri* serotypes X and 2 were the most common at 10.61% and 8.33%, respectively (Fig. 1). None of the samples tested positive for *S. flexneri* serotype 4.

### AMR genes

AMR genes from multiple classes were harboured in 51 (85%) of the 60 samples positive for *S. flexneri* and *S. sonnei*. The *mphA* gene, which confers resistance to macrolides, was detected in 98.3% (59/60) of samples positive for *S. flexneri* and *S. sonnei*. The β-lactamase gene *CTX-M1-9* was present in 85% (51/60) of these samples. Additionally, the *CTX-M2-8-25* gene was identified in 26.7% (16/60) of the positive samples. Only four samples (6.7%) tested positive for the New Delhi metallo-β-lacatamase (*NDM)* gene, and none harboured the Mobilized colistin resistance (*MCR)* gene, which confers resistance to polymyxin.

### Clinical, socioeconomic and behavioural factors of *Shigella*-associated diarrhoea

The majority of the participants presented with fever (290, 75.13%) and no dehydration (284, 73.58%) while 14 (3.63%) presented with bloody diarrhoea. The proportion of children presenting with bloody diarrhoea was higher among children above two years (57.14%, 8/14). Bivariate analysis showed that *S. flexneri* and *S. sonnei* positivity was significantly associated with abdominal pain (COR = 2.169, 95% CI 1.11–4.26, *p* = 0.024) and headache (COR = 2.13, 95% CI 1.08–4.24, *p* = 0.030) (Table 4). Multivariable analysis, adjusting for age and other relevant variables, showed that positivity for *S. flexneri* and *S. sonnei* was not significantly associated with abdominal pain or headache, suggesting that these symptoms are not independent predictors of infection in this population (Table 6). Multivariable analysis showed that vomiting was significantly associated with lower odds of *S. flexneri* or *S. sonnei* detection (aOR = 0.47, 95% CI 0.25–0.86, *p* = 0.014), indicating a 53% reduction in odds compared to those without vomiting.

**Table 4:** Clinical characteristics associated with *S. flexneri* and *S. sonnei* detection among children under five years in urban Kenya

Variable	Categories	Negative f(%)	Positive f(%)	COR	*p*-value	95% CI
Fever	No	79 (82.29)	17 (17.71)	Ref	-	-
	Yes	247 (85.17)	43 (14.83)	0.809	0.5	0.4369–1.4979
Continuous fever	No	140 (84.34)	26 (15.66)	Ref	-	-
	Yes	107 (86.29)	17 (13.71)	0.855	0.644	0.4417–1.6570
Number of days	1	26 (81.25)	6 (18.75)	Ref	-	-
	2	25 (83.33)	5 (16.67)	0.867	0.83	0.2344–3.2045
	3	43 (87.76)	6 (12.24)	0.605	0.423	0.1764–2.0726
	4	10 (100.00)	0 (0.00)	-	-	-
	5	1 (100.00)	0 (0.00)	-	-	-
	7	2 (100.00)	0 (0.00)	-	-	-
Vomiting	Don’t Know	1 (33.33)	2 (66.67)	7.23	0.112	0.631–82.91
	No	94 (78.33)	26 (21.67)	Ref	-	-
	Yes	231 (87.83)	32 (12.17)	0.500	0.017	0.283–0.885
Abdominal pain	Don’t Know	16 (100.00)	0 (0.00)	-	-	-
	No	109 (90.08)	12 (9.92)	Ref	-	-
	Yes	201 (80.72)	48 (19.28)	2.169	0.024	1.105–4.256
Diarrhoea	No	8 (88.89)	1 (11.11)	Ref	-	-
	Yes	318 (84.35)	59 (15.65)	1.484	0.712	0.1822–12.0886
Bloody diarrhoea	No	316 (84.95)	56 (15.05)	Ref	-	-
	Yes	10 (71.43)	4 (28.57)	2.257	0.181	0.6840–7.4480
Headache	Don’t Know	66 (94.29)	4 (5.71)	0.328	0.040	0.113–0.950
	No	222 (84.41)	41 (15.59)	Ref	-	-
	Yes	38 (71.70)	15 (28.30)	2.13	0.030	1.078–4.236
Dehydration	Mild	71 (80.68)	17 (19.32)	Ref	-	-
	Moderate	10 (90.91)	1 (9.09)	0.418	0.42	0.0500–3.4887
	None	242 (85.21)	42 (14.79)	0.725	0.311	0.3890–1.3507
	Severe	3 (100.00)	0 (0.00)	-	-	-

*COR, crude odds ratio; CI, confidence interval

Most of the guardians (77.72%, 300/386) had attained at least secondary-level education. Most households comprised more than four members (51.30%, 198/386) and resided in permanent single-room dwellings (Table 5). There was no significant association between households that use communal or municipal water and *S. flexneri* or *S. sonnei* detection in children under five years (COR = 0.17, 95% CI 0.01–2.86, *p* = 0.22) (Table 5). Participants who reported treating their water had slightly lower odds of *S. flexneri* or *S. sonnei* detection compared to those who did not treat their water, with an odds ratio (OR) of 0.89. However, this association was not statistically significant (*p* = 0.686), and the 95% confidence interval (CI: 0.51–1.55) includes 1, indicating no clear evidence of a protective effect. Among participants who treated their water, boiling was the most common method (63.4%), followed by use of Aquatabs^®^ (20.9%) and filtration (15.7%). The presence of environmental contamination—such as open sewer systems or visibly polluted surface water—within 20 m of a household water source was not significantly associated with an increased risk of *S. flexneri* or *S. sonnei* detection. (COR = 1.72, 95% CI 0.81–3.66, *p* = 0.157) (Table 5). Bivariate analysis revealed that *S. flexneri* or *S. sonnei* detection among children in Mukuru informal settlement was significantly associated with obtaining vegetables from the market (COR = 2.62, 95% CI 1.08–6.36, *p* = 0.03) (Table 5). Some variables that were significant in the crude analysis, such as age in months and obtaining vegetables from the market, remained significant after adjustment (Table 6).

**Table 5:** Demographic, socioeconomic, hygiene, and behavioural factors associated with *S. flexneri* and *S. sonnei* detection among children in urban Kenya

Variable	Categories	Negative f (%)	Positive f (%)	COR	*p*-value	95% CI
Education	Primary and Below	73 (84.88)	13 (15.12)	ref		
	Secondary and Above	253 (84.33)	47 (15.67)	1.043	0.901	0.535–2.033
No. of family members	2–3	157 (83.51)	31 (16.49)	ref		
	4 and above	169 (85.35)	29 (14.65)	0.869	0.618	0.501–1.508
No. of Children < 5years	1	275 (83.59)	54 (16.41)	ref		
	2	45 (88.24)	6 (11.76)	0.679	0.399	0.276–1.671
	3	5 (100.00)	0 (0.00)	-	-	-
	4	1 (100.00)	0 (0.00)	-	-	-
House type	Makeshift	151 (82.51)	32 (17.49)	ref		
	Permanent	175 (86.21)	28 (13.79)	0.755	0.318	0.435–1.311
No. of Rooms	One	277 (83.94)	53 (16.06)	ref		
	Two	32 (84.21)	6 (15.79)	0.98	0.966	0.391–2.459
	Three and Above	17 (94.44)	1 (5.56)	0.307	0.257	0.040–2.360
Main source of water	Borehole	1 (50.00)	1 (50.00)	Ref	-	-
	Communal well/pump	1 (50.00)	1 (50.00)	1	1	0.02–50.39
	Communal/Municipal tap	150 (85.23)	26 (14.77)	0.17	0.22	0.01–2.86
	Own tap	6 (85.71)	1 (14.29)	0.17	0.314	0.01–5.45
	Water Vendor	13 (86.67)	2 (13.33)	0.15	0.244	0.01–3.57
	Bottled water	27 (87.10)	4 (12.90)	0.15	0.207	0.007–2.87
Generally treat water	No	181 (83.80)	35 (16.20)	Ref	-	-
	Yes	145 (85.29)	25 (14.71)	0.89	0.686	0.51–1.55
Treatment by boiling	No	71 (83.53)	14 (16.47)	Ref	-	-
	Yes	74 (87.06)	11 (12.94)	0.75	0.517	0.32–1.77
Treatment by water guard or aqua tabs	No	72 (91.14)	7 (8.86)	Ref	-	-
	Yes	22 (78.57)	6 (21.43)	2.8	0.089	0.85–9.22
Treatment by filtration	No	74 (86.05)	12 (13.95)	Ref	-	-
	Yes	20 (95.24)	1 (4.76)	0.31	0.272	0.04–2.51
Sources of contamination	No	76 (89.41)	9 (10.59)	Ref	-	-
	Yes	250 (83.06)	51 (16.94)	1.72	0.157	0.81–3.66
Wash hands after visiting toilet	Always	214 (83.27)	43 (16.73)	Ref	-	-
	Never	1 (100.00)	0 (0.00)	-	-	-
	Sometimes	111 (86.72)	17 (13.28)	0.76	0.38	0.41–1.40
Cook vegetables without washing	No	98 (84.48)	18 (15.52)	Ref	-	-
	Yes	100 (85.47)	17 (14.53)	0.93	0.833	0.45–1.90
Obtain vegetables from the market	No	308(85.56)	52(14.44)			
	Yes	18(69.23)	8(30.77)	2.632	0.032	1.08–6.36

*COR, crude odds ratio; CI, confidence interval; WG, WaterGuard^®^ (Population Services International, Nairobi, Kenya); AT, AquaTabs^®^ (Medentech, Wexford, Ireland)

**Table 6:** Multivariable logistic regression analysis of factors associated with *S. flexneri* and *S. sonnei* detection among children in urban Kenya

Variable	Category	aOR	95% CI	*p*-value
Age (months)	Continuous	1.025	1.003–1.047	0.027
Vomiting	No / Don’t Know	1 (ref)	–	–
	Yes	0.474	0.264–0.851	0.012
Headache	No / Don’t Know	1 (ref)	–	–
	Yes	1.594	0.763–3.331	0.215
Abdominal pain	No / Don’t Know	1 (ref)	–	–
	Yes	1.665	0.784–3.538	0.185
Obtain vegetables from the market	No	1 (ref)	–	–
	Yes	2.737	1.081–6.930	0.034

*aOR, adjusted odds ratio; CI confidence interval

## Discussion

Our study demonstrated a high burden of shigellosis due to S. *flexneri* and *S. sonnei* in a densely populated urban slum in Kenya. The prevalence of *S. flexneri* and *S. sonnei* among diarrheic children under five years was 15.54%. A study conducted in Guinea-Bissau reported a *Shigella* prevalence of 27.2% in a similar target group [25]. The prevalence observed in this study was comparable to that reported in a study conducted in Rwanda among a similar population, which documented a detection rate of 13% [26]. Notably, all three studies employed PCR for pathogen detection. The prevalence of *Shigella* spp., as determined by culture, was 1.5% in children under five years of age in Mukuru and 11.05% in Kibera, two urban informal settlements in Kenya [9, 10]. In the first study, the isolation rate of *S. flexneri* across different age groups was 59.7%. In the second study, *S. flexneri* accounted for 64% of isolates, while *S. sonnei* comprised 9%. Similarly, a study from Ethiopia that utilized culture-based methods among children under five years of age reported a *Shigella* prevalence of 13.3% [27]. In the VIDA study, *S. flexneri* accounted for 67.6% of isolates, while *S. sonnei* represented 18.2% [8]. The pooled prevalence of *Shigella* among children under five years of age across five South Asian countries, based on studies employing various diagnostic methods, was 10% [28].The variation in prevalence may be attributed to differences in geographical locations and varying diagnostic methods employed, with PCR offering greater sensitivity and specificity compared to conventional culture techniques.

*S. flexneri* was the most common species and this agrees with other studies done in the country and other LMICs [2, 9, 10, 21, 28–32]. *S. flexneri* serotypes 2,3,5,6 and X were the most common serotypes. Nonetheless, the detection rate of serotype X in the present study was comparatively higher than that observed in the earlier studies. This finding aligns with the report by M.H. Ahmed et al.., suggesting a global rise in the detection of serotype X and highlighting its role—along with its variants—in notable outbreaks reported in China [33, 34]. Our findings align with those reported by Elizabeth et al., who observed serotypes 2a and 6 as the most common across multiple countries [35], as well as with patterns described in the Global Enteric Multicenter Study (GEMS).Evidence from a systematic review spanning five South Asian countries demonstrated that serotype 2a is the predominant circulating *S. flexneri* serotype, followed by serotypes 3a, 6, and 1b [28]. In contrast to the VIDA *Shigella* study, in which *S. flexneri* serotype 4a was detected at a rate of 5.1%, the serotype was not identified in our study [8]. Like the Jie Liu et al. study, mixed *S. flexneri* serotype infections were also identified in the study population [35]. Some *Shigella* serotypes share antigens that may provide cross-protection, making it essential to understand serotype profiles to guide vaccine development. Additionally, continuous serotype surveillance is crucial for accurately identifying circulating strains and supporting targeted prevention strategies [16].

The macrolide azithromycin is recommended by the American Academy of Pediatrics for the treatment of shigellosis in children [36, 37]. However, recent reports have documented the emergence of *Shigella* isolates with reduced susceptibility to azithromycin, which has been linked to the presence of plasmid-mediated *mphA* and/or *ermB* genes [36, 38]. In this study, several key AMR genes were identified in stool samples positive for *S. flexneri* and/or *S. sonnei*. In our study, 98.3% of the tested samples were positive for the *mphA* gene. Comparable findings have been reported in Bangladesh, where 95% of 37 azithromycin-resistant *Shigella* isolates carried the *mphA* gene [39]. In Kenya, a study conducted in an urban informal settlement found that over half of the *S. flexneri* isolates (22, 55%) exhibited intermediate resistance to azithromycin, as did 14 (51.9%) of the *S. sonnei* strains. The rapid spread of macrolide resistance genes among *Shigella* is driven by horizontal gene transfer [39]. As enteric bacteria interact within the gut and could exchange resistance genes [40], prudent use of antimicrobials is essential to limit the emergence and dissemination of resistant strains. Widespread antibiotic use has contributed to the emergence of resistance to multiple antibiotic classes, including β-lactams such as cephalosporins and carbapenems [41]. The emergence of extended-spectrum β-lactamases (ESBLs) in *Shigella* species represents a significant global public health threat, impacting both developed and developing countries [10]. Most samples tested positive for the *CTX-M* gene, a member of the ESBLs, which confer resistance to third-generation cephalosporins. The high detection rate of the *CTX-M* gene is consistent with findings from a study conducted in Iran on *Shigella* isolates obtained from pediatric cases [11]. *CTX-M* was predominantly found in *S. flexneri*-positive samples. The identification of ESBL in *Shigella* positive samples is a significant public health concern, as it could limit the available antibiotic treatment options. These findings underscore the need for continuous surveillance of antimicrobial resistance to inform empiric treatment strategies and improve patient management outcomes. The *NDM-1* gene, a metallo-β-lactamase that confers resistance to carbapenems (a subclass of β-lactams often used as last-resort antibiotics), was detected in only four samples. The gene is frequently plasmid-borne, facilitating its transfer to other microorganisms through horizontal gene transfer, and thereby promoting the emergence of drug-resistant pathogenic strains [42], and was detected in both *S. flexneri* and *S. sonnei* serogroups.

The finding that age was a significant risk factor that predisposes children under five years to *Shigella* associated diarrhoea (aOR = 1.025, 95% CI: 1.003–1.047, *P* = 0.027) is similar to findings from a study conducted previously in Kenya [43]. These findings are in agreement with Zachariah et al. (2021), who observed an age-related increase in *Shigella* isolation in a facility-based study in Kapsabet, Kenya, with the highest rates seen in children aged 12–23 months. Similarly, a study in Ethiopia and other countries revealed a high prevalence of *Shigella* in the age group 12–23 months [27, 31, 44, 45]. The observed pattern may be attributed to infants’ immature immune systems and increased pathogen exposure through behaviours like mouthing during early development [46]. In contrast, children under one year may benefit from the protective effects of breast milk and limited exposure to contaminated food [47]. The rise in shigellosis cases after infancy likely reflects waning maternal antibody protection and insufficient acquired immunity [48]. The majority of the participants who tested positive for *S. flexneri or S. sonnei* and had bloody stools were from the oldest age group, which is consistent with the VIDA Study [8]. The crude data in this study identified that the clinical presentation of diarrhoea associated with *S. flexneri* and/or *S. sonnei* commonly included vomiting and abdominal pain. These study findings partially agree with Patricia et al. study where the clinical presentation of *Shigella* in infants commonly included vomiting, dehydration, and absence of dysentery [49].

There was no significant association between the guardians’ level of education and *Shigella*-associated diarrhoea in contrast to previous studies by Ongadi et al. and Ugboko et al. [10, 48]. Literacy, particularly among women, has consistently been identified as a key determinant of health outcomes across populations. Higher levels of female education are associated with improved knowledge and practices related to personal hygiene, nutrition, and more effective utilization of healthcare services [48].

Notably, sourcing vegetables from local markets suggests a potential risk factor for diarrhoea associated with *S. flexneri* and/or *S. sonnei* (aOR = 2.737, 95% CI: 1.081–6.930, *P* = 0.034). While this may represent a more economically accessible option for households, it underscores the need for targeted public health education on proper vegetable washing and safe food handling practices prior to consumption.

One limitation of this study is its reliance on the healthcare-seeking behavior of individual patients. Cases involving children with diarrhoea who self-medicated, used home-based treatments, or sought care from alternative healthcare facilities may have been missed. Additionally, although *Shigella dysenteriae* and *Shigella boydii* are uncommon in our setting, including these serogroups in the TAC panel could have offered additional insights into the local epidemiology of *Shigella* infections. While we detected the presence of AMR genes in *Shigella* positive fecal samples, we cannot infer that these are associated with phenotypic AMR in *Shigella*. PCR detects DNA and does not distinguish between viable and non-viable pathogens; therefore, positive results may reflect DNA from both live and dead organisms. Given the cross-sectional nature of this study, causality cannot be inferred. The findings only demonstrate associations between variables. Further longitudinal studies are needed to explore potential causal pathways underlying this association.

In conclusion, this study reveals a significant burden of *S. flexneri* and *S. sonnei* among children under the age of five years. PCR proved more sensitive than culture, highlighting the value of molecular diagnostics in surveillance. The high prevalence of ESBL genes, particularly *CTX-M*, the detection of *NDM-1*, and the presence of the *mphA* gene raise significant concerns about the growing burden of antibiotic resistance. These findings underscore the need for improved hygiene practices, strengthened AMR surveillance, antimicrobial stewardship, and targeted public health interventions.

## Data Availability

Data are available from the corresponding author on reasonable request and with approval from the institutional Review Board.
